# Sleep-Related Declarative Memory Consolidation and Verbal Replay during Sleep Talking in Patients with REM Sleep Behavior Disorder

**DOI:** 10.1371/journal.pone.0083352

**Published:** 2013-12-13

**Authors:** Ginevra Uguccioni, Olivier Pallanca, Jean-Louis Golmard, Pauline Dodet, Bastien Herlin, Smaranda Leu-Semenescu, Isabelle Arnulf

**Affiliations:** 1 Sleep Disorder Unit, Pitié-Salpêtrière University Hospital, Assistance Publique-Hôpitaux de Paris, Paris, France; 2 Centre de Recherche de l’Institut Cerveau-Moelle épinière, Institut national de la santé et de la recherche médicale Unité 975, Pierre and Marie Curie University ; Institut national de la santé et de la recherche médicale Unités mixtes de recherches 975, Centre national de la recherche scientifique Unités mixtes de recherche 7225, Paris, France; 3 Department of Biostatics, Pitié-Salpêtrière Hospital, ER4, Paris 6 University, Paris, France; 4 Institut National de la Santé et de la Recherché Médicale, Unité 975, Paris, France; Charité University Medicine Berlin, Germany

## Abstract

**Objective:**

To determine if sleep talkers with REM sleep behavior disorder (RBD) would utter during REM sleep sentences learned before sleep, and to evaluate their verbal memory consolidation during sleep.

**Methods:**

Eighteen patients with RBD and 10 controls performed two verbal memory tasks (16 words from the Free and Cued Selective Reminding Test and a 220-263 word long modified Story Recall Test) in the evening, followed by nocturnal video-polysomnography and morning recall (night-time consolidation). In 9 patients with RBD, daytime consolidation (morning learning/recall, evening recall) was also evaluated with the modified Story Recall Test in a cross-over order. Two RBD patients with dementia were studied separately. Sleep talking was recorded using video-polysomnography, and the utterances were compared to the studied texts by two external judges.

**Results:**

Sleep-related verbal memory consolidation was maintained in patients with RBD (+24±36% words) as in controls (+9±18%, *p*=0.3). The two demented patients with RBD also exhibited excellent nighttime consolidation. The post-sleep performance was unrelated to the sleep measures (including continuity, stages, fragmentation and apnea-hypopnea index). Daytime consolidation (-9±19%) was worse than night-time consolidation (+29±45%, *p*=0.03) in the subgroup of 9 patients with RBD. Eleven patients with RBD spoke during REM sleep and pronounced a median of 20 words, which represented 0.0003% of sleep with spoken language. A single patient uttered a sentence that was judged to be semantically (but not literally) related to the text learned before sleep.

**Conclusion:**

Verbal declarative memory normally consolidates during sleep in patients with RBD. The incorporation of learned material within REM sleep-associated sleep talking in one patient (unbeknownst to himself) at the semantic level suggests a replay at a highly cognitive creative level.

## Introduction

After a learning episode, still fragile memory traces are progressively converted over time into more stable representations in long-term memory [1]. This memory consolidation, or the process of trace stabilization and strengthening, preferentially occurs during sleep [2–4]. According to the two-stage model of memory, events experienced during wakefulness are initially encoded in parallel in neocortical networks and in the hippocampus. During subsequent sleep, the newly acquired memory traces are repeatedly reactivated and thereby become gradually redistributed such that synaptic connections within the neocortex are strengthened forming more persistent memory representations (see a review in [5]). Procedural memory predominantly benefits from rapid eye movement (REM) sleep whereas hippocampus-dependent declarative memory benefits particularly from non REM sleep. Post-learning sleep benefits to verbal declarative memory [6,7], emotional memory [8,9], spatial learning [10], and motor procedural learning [11]. Consequently, interrupted sleep and sleep disorders may result in cognitive deficits. Young patients with mild sleep apnea perform worse at a motor sequence task that they trained on in the evening after a night sleep compared to healthy controls, a decreased performance that correlates with sleep fragmentation [12]. Patients with primary insomnia demonstrated a lower sleep-associated memory consolidation of declarative (verbal and visual memory) memory tasks, while results are more controversial for procedural memory (depending on the test) compared to good sleepers [13]. Patients with narcolepsy show less consolidation of a visual procedural (texture discrimination) task after sleep compared to controls [14]. However, it is not known how parasomnias (including sleepwalking and REM sleep behavior disorder) affect sleep-dependent memory consolidation. 

Parasomnias may also be original models to understand the mechanisms of sleep-dependant memory consolidation. In addition to passively protecting recent memory traces from interferences, sleep appears to exert active mechanisms on memory. The main hypothesis states that the neural traces encoding newly acquired information are reshaped and strengthened via reactivation processes during sleep. In rats, specific patterns of neural activity associated with recent waking behavior of spatial navigation are spontaneously replayed during subsequent sleep [15]. In birds, the timing and structure of activity in neurons of the motor cortex elicited by the playback of a song during sleep matches the activity observed during daytime singing [16]. Moreover, the “spontaneous” activity of these neurons during sleep matches their sensorimotor activity, a form of song “replay”. Similarly, brain regions involved in motor skill learning are reactivated during post-training sleep in human function imaging studies [17]. These regional reperfusions could correspond (as observed in animals) to the replay during sleep of temporally-organized patterns of neural activity encoding for newly acquired information, or they may simply reflect other experience-dependent brain processes, such as local homeostasis [18]. 

We recently addressed this question by studying patients who exhibited overt dreamlike behaviors during two types of parasomnias, non-REM sleep (sleepwalkers) and during REM sleep (REM sleep behavior disorder, RBD) [19]. We intensively trained sleepwalkers and patients with RBD during the evening with a serial reaction time task. Without explicitly knowing it, the subjects learned specific hand and arm choreography. We found that both patient groups improved their motor performance after sleep. One of the sleepwalkers partially re-enacted the learned movements during non REM sleep, providing the first evidence of a temporally-structured replay of a learned behavior during sleep in humans. Here we aimed to repeat this experiment using declarative verbal learning. Indeed, patients with RBD frequently talk during REM sleep. Chronic RBD affects middle-aged men, as an isolated condition (idiopathic RBD) or in association with neurodegenerative diseases (mainly synucleinopathies) [20]. As a consequence, the patients frequently demonstrate concomitant mild cognitive impairment, despite non REM sleep (and particularly N3 sleep, also called slow-wave sleep) is normal in idiopathic RBD [21] as well as in RBD associated with Parkinson’s disease [22]. Our first goal was to determine if the patients could replay words or sentences learned before sleeping while sleep talking during REM sleep, using this unique model of RBD with sleep-associated speeches. Our second goal was to determine if patients with RBD maintain their ability to consolidate verbal memory during sleep despite the RBD disorder and cognitive impairment. 

## Subjects and Methods

### 1: Ethic statement

The institutional review board 01-10 of the ethics committee (Comité de Protection des Personnes Ile de France 06) gave its approval for the study, which was considered as non invasive. The patient has given written informed consent, as outlined in the PLOS consent form, to publication of their video.

### 2: Participants

We consecutively recruited patients referred to the sleep disorder unit of a university hospital for suspected REM behavior disorder (RBD) from September 2011 to November 2012. RBD diagnosis was confirmed by clinical interview and video-polysomnography in all patients. The patients met the international criteria for RBD, including: (i) a clinical history of complex, vigorous, violent or injurious behavior during sleep frequently associated with dream mentation; plus (ii) enhanced chin muscle tone during REM sleep; or (iii) simple or complex behaviors on video during REM sleep [23]. We included patients with idiopathic RBD as well as patients with RBD associated to Parkinson’s disease, multiple systemic atrophy and possible Lewy body dementia. The patients’ cognitive status was measured using the Montreal Cognitive Assessment (MoCA) [24] which detects mild cognitive impairment earlier than the Mini-Mental Status Examination in patients with RBD [25]. Patients with a score below 26/30 were considered to have mild cognitive impairment, and patients with a score below 23/30 were diagnosed with dementia (Lewy body dementia).

In addition, control adults were recruited from the same sleep laboratory among healthy snorers or subjects complaining of difficulties in initiating sleep. These subjects were referred to the sleep laboratory for suspicion of concomitant sleep apnea, but they were eventually diagnosed with normal sleep (apnea-hypopnea index below 15 without any symptom of sleep apnea syndrome) and underwent the same procedure. To be included in the study, the subjects had to meet the following criteria: (i) no final diagnosis of any sleep disorder; (ii) absence of mild cognitive impairment defined as MoCA greater than or equal to 27; (iii) absence of neurological disease; and (iv) absence of RBD and pre-RBD defined as a percentage of REM sleep without atonia lower than 15% [26]. These subjects were carefully matched to the RBD patients for age, gender and years of education.

The participants provided written consent prior to the start of the learning procedure. The study protocol (including the emotionally balanced texts) was approved by the local Ethics committee (Comité de Protection des Personnes Ile de France-06). 

### 3: Clinical interview and psychological scales

All the patients underwent a semi-structured interview that included an assessment of the clinical history of the disease, age at onset and family history. The patients completed two self-administered questionnaires, including a questionnaire on hospital anxiety and depression rating scale (HAD) [27] and an aggression questionnaire (AQ) [28].

### 4: Experimental task

Two verbal memory tasks were performed before night-time sleep, including the Free and Cued Selective Reminding Test [29] and the modified Story Recall Test. Verbal memory was evaluated using the Free and Cued Selective Reminding Test, which is a memory task that controls attention and strategy and is used to maximize learning. This test provides a measurement of short-term memory that is not confounded by deficits in other cognitive abilities. Briefly, a list of 16 words is encoded and supported by semantic cueing. The 16 to-be-learned words belong to 16 different semantic categories and were presented to the subject on four different cards, where each card contained four words. “Verbal encoding” was performed one card at a time. The subject was asked to point to and read aloud each word when its category cue was verbally provided. The card was then removed and the encoding was tested by providing the category cue for each of the four words. The “verbal encoding score” (from 0 to 16) was determined by the number of words that were correctly retrieved on the first trial at immediate cued recall. The recall phase of the 16 words included three trials. Each trial consisted of an extended period of free recall (up to 2 minutes), which was immediately followed by a cued recall for items that was not retrieved during free recall. The verbal learning free recall score (from 0 to 48) was determined by the total number of words that were correctly evoked on the three successive trials. The verbal learning total recall score (from 0 to 48) was the number of words that were correctly evoked at free or cued recall. The delayed verbal free recall and delayed verbal total recall scores were also measured after a 20-minute period. Sensitivity to the verbal cue index measured the proportion of semantic cues that triggered correct responses. 

The stories to be learned were obtained from the news in brief articles published in French newspapers. These articles were selected for their intense emotionally negative content (see Annex 1), because emotional items are more strongly consolidated across sleep than neutral items [30]. The first story (A Love to Death, 220 words) referred to a mother that wandered the streets of Chicago with her baby and who killed him at the end of the story. The second story (Cannibalism, 263 words) described a woman eating her lover after cutting him into pieces. The subjects were asked to learn the story by heart (memorize). They then completed 12 graduated Likert adjective scales with 7 grades (-3, -2,-1, 0, 1, 2, 3) based on what they felt and thought about the text, including “comprehensible vs. incomprehensible for you”, “interesting vs. not interesting for you”, “difficult vs. easy to learn”, “emotional vs. neutral for you”, “positive vs. negative general emotion of the text”, “fact already know vs. unknown from you”, “ribald vs. terrifying facts”, “important vs. not important information, for you”, “ concrete or abstract words for you”, “funny vs. serious facts for you”, “boring vs. exciting facts for you”, or “lay people words or not”. The psychologist explained the adjective senses if the subjects asked. 

### 5: Experimental cognitive procedure

**Figure 1:**
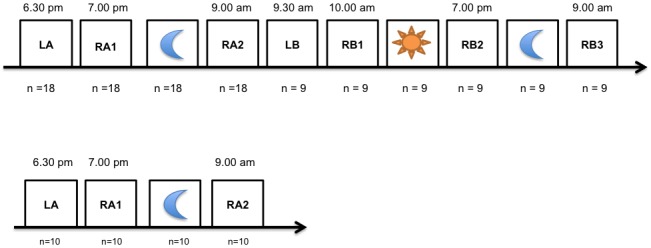
Schematic design of the cross-over randomized trial for patients with REM sleep behavior disorder (upper panel) and control subjects (lower panel). **LA:** learning of FCSRT and first Story Test ; **RA1/RA2**: recall of FCSRT and first Story Test ; **LB:** learning of second Story Test ; **RB1/RB2/RB3**: recall of second Story Test .

A schematic of the study design of the cross-over trial is shown in Figure 1. The order of story texts was balanced one after one (e.g., AB in Subject 1, then BA in Subject 2). The list of 16 words from the FCSRT was first encoded (immediate free and cued recall). To fill the 20-minute period between encoding and long term recall, the subjects completed the HADRS, the AQ and silently read the two texts (one neutral story N on bank stock options, which contained 155 words, and one positive story P on the reunion of a child and his mother after 20 years of separation, containing 332 words), and then scored their feelings after reading the two texts using the 12 Likert scale. The aims of this method were to calibrate the emotional aspects of the texts and to avoid having the subject practice the 16 words before recall. The long-term free and total (cued) recall of the FCSRT was obtained. Next the subjects encoded the modified Story Recall Test A or B (the order was randomized 1:1) between 7:00 and 7:30 pm, and were asked to memorize what they learned. The first oral retrieval of the RA1 text occurred after this 30-min learning session. It was immediately transcribed word-for-word on a sheet by the psychologist. The text sheet was removed, and the subjects were advised to mentally recall the text before sleeping for testing the next morning. 

Video Polysomnography was performed on the subjects at night beginning *ad libitum* (with a last limit of 11:00 pm) and ending *ad libitum* (with a last limit of 08:45 am). The retrievals of the FCSRT and story text (RA2) were tested at 9:00 am in all subjects (night-time consolidation group, after a duration of 11hours and 30 min, which included sleep time). A subgroup of 9 patients with RBD had a daytime learning LB in the morning between 9:30 am and 10 am of the second story Text B (n=4 5), or A (n=5) depending on the cross-over design. This component of daytime consolidation was not tested in the control subjects. The 9 patients with RBD had a second retrieval (RB2) after one day of wakefulness in the absence of any sleep (daytime consolidation, lasting 9 hours). Video-polysomnography was performed on these 9 patients with RBD on the second night in the sleep laboratory, and the patients were retested for the second text retrieval (RB3) at 9 am on the third day to evaluate the 24-hr consolidation. 

### 6: Video-polysomnography

Video polysomnography was performed to evaluate the sleep quality and quantity in all subjects and to test the hypothesis of verbal replay in the RBD group. For this purpose, video-polysomnography was performed during one night on 18 patients with RBD and on 10 controls subjects. In addition, video-polysomnography was performed on10 patients with RBD on the second night (on the following night since replay might occur over several nights). The video and sleep monitoring included Fp1-Cz, O2-Cz, and C3-A2 electroencephalography, a right and left electro-oculogram, EMG of the levator menti and tibialis anterior muscles, nasal pressure through a cannula, tracheal sounds through a microphone, thoracic and abdominal belts to assess respiratory efforts, electrocardiography, pulse oximetry, as well as EEG-synchronized infrared video-monitoring and ambiance microphone. The sleep stages [31], arousals, respiratory events, periodic leg movements and muscle activities during REM sleep were scored using visual inspection according to international criteria [32]. The spindle index (number of spindles per min of stage N2) was manually measured by visually counting sleep spindles (figures lasting 0.3 to 1 s with a frequency of 12 to 15 Hz) during 10 min of uninterrupted N2 stage during the first sleep cycle. REM sleep without atonia was measured on the chin EMG, following the international rule, and expressed as the percentage of REM epochs with more than half of enhanced muscle tone divided by the total REM sleep epochs [32]. In addition, we examined the night video and audio recordings and selected the verbal utterances (sleep talking) obtained during REM sleep (nothing was found during non REM sleep) in patients with RBD. The video clips were stored and translated into written sentences. This translation was performed by two independent scorers who discussed and reviewed any discrepancies. The sleep talking material was compared to the learned verbal material (FCSR and story texts) by two independent judges to assess the identity and similarity of the meaning. 

### 7: Data management and statistical analysis

The retrieved texts were quantified for the number of identical words from the learned texts. The consolidation percentage was obtained by subtracting the number of recall words from the total number of to-be-learned words in the text, the result of which was divided by the total number of to-be-learned words in the text for each subject. Because there were two texts, the final immediate, post-night, and post-day consolidations were measured by pooling these percentages. 

Two sets of statistical analysis were performed, one including all of the participants, and the other excluding patients with dementia. These patients were not completely excluded because we aimed at testing whether a patient could consolidate memory during sleep despite Lewy body dementia, in contrast with patients with mild Alzheimer disease who have impaired sleep-related consolidation of episodic memory and reduced fast sleep spindles [33]. To display homogeneous groups, we only provided the mean, standard deviation, median, interquartile intervals, confidence intervals and medians excluding the dementia patients, in this article. Comparisons between groups were performed using the Wilcoxon test, with a significance of p < 0.05. We also compared night-time and day-time consolidation on the sub-group of 9 patients with RBD who have had day-time consolidation using the paired-Wilcoxon test, with a significance of p < 0.05. The sleep-related changes in performance were correlated with all sleep measures using the Spearman test. All statistics were performed using the SAS software SAS Institute, Cary, NC. 

## Results

### 1: Psychological characteristics of the subjects during wakefulness

There were 20 patients with RBD and 10 control subjects in this study. Two patients with RBD had a MoCa score of less than 23, including one patient with concomitant Parkinson’s disease dementia (MoCa score: 22) and a patient with Lewy body dementia (Moca score: 20). These patients were excluded from the statistical analysis. The final sample included 18 patients with RBD and 10 control subjects. The patients had idiopathic RBD (n=9), and RBD associated with Parkinson disease (n=7), as well as multiple systemic atrophy (n=2). Expectedly, using the matching process, there were no differences in age, sex or level of education between the patients with RBD and the control subjects (Table 1). In addition, the groups did not differ in their levels of subjective daytime sleepiness, aggressiveness during the daytime (evaluated on the AQ scale) and scores of depression and anxiety. 

**Table 1:** Clinical characteristics and psychological scales of patients with REM sleep.

Patients	**REM sleep behavior disorder**	**Controls**	**p-value**
Number of subjects	18	10	
Age, y	67±7.6	64.2±9.3	0.27
Sex, % women	33.3	50	0.44
Education levels, 1-7	4.7±2.1	5.3±1.6	0.39
Epworth sleepiness score, 0-24	8.6±5	8±5.5	0.96
Aggression questionnaire, 29-145	66.1±19.7	64±19	0.73
Physical aggression, 9-45	18.5±7.8	15.5±5.6	0.44
Verbal aggression, 5-25	12.1±4.6	12.9±4	0.60
Anger, 7-35	16.8±6.3	17.6±5.2	0.58
Hostility, 8-40	18.9±7.3	18.1±6.8	0.80
HAD total	17.4±7.7	15.5±8	0.51
Anxiety subscore	10.6±3.6	9.2±4	0.30
Depression subscore	6.8±4.7	6.3±4.8	0.76

HAD: Hospital Anxiety and Depression Scale. Data were expressed as the mean±SD

### 2: Sleep measures in patients with RBD and the control subjects

The total sleep time was similar between the two groups; however, patients with RBD had higher N1 and lower N3 sleep stage percentages compared to the controls (Table 2). The indices of sleep continuity (sleep onset latency, sleep efficiency, wakefulness after sleep onset) and fragmentation were not different between the groups, as well as the spindle index. Notably, the two patients with RBD plus dementia had no spindle activity. 

**Table 2:** Sleep measures in patients with REM sleep behavior disorder and controls.

Subjects	**REM sleep behavior disorder**	**Controls**	**p-value**
Number of subjects	18	10	
Latency to, min			
Sleep onset	43±31	32±37	0.32
REM sleep	156±115	142±87	0.98
Sleep efficiency, %	76±14.5	84.4±11	0.10
Wakefulness after sleep onset, min	124±79	79±65	0.14
Total sleep time, min	397±97	417±82	0.66
Sleep stage, % of total sleep time			
N1, %	9.2±5.6	4.8±2.8	**0.04**
N2, %	53.8±12	53.1±9.8	0.88
N3, %	18±8.3	24.6±6.5	**0.02**
REM, %	18.5±7.2	14.9±5.5	0.25
REM sleep without atonia, % of total REM sleep	48.3±34.4	0.5±1.6	**0.0005**
Sleep spindles, n/min	2.5±2.2	3.3±2.5	0.47
Sleep fragmentation, n/h			
Arousals	10.6±8.3	8.5±4.8	0.47
Periodic leg movements	0.9±1.4	1±2.2	0.75
Apnea hypopnea	12.6±11	9.3±6.8	0.82

Data were expressed as the mean±SD

Expectedly, patients with RBD had higher percentage of REM sleep without atonia (range: 0-100%) compared to the controls (range: 0-5%). Moreover, the control subjects demonstrated no motor behavior or verbal utterances during sleep, while patients with RBD had simple or complex movements (including the patient with RBD and 0% of REM sleep with atonia). Eleven patients with RBD spoke during REM sleep.

### 3: Cognition tests before sleep

For cognition (Table 3), the mean MoCa scores were lower in patients with RBD (range: 23-28) compared to controls (range: 27-30). The scores of the verbal encoding and immediate free recall (individual, over 16 and total, over 48) were not different in RBD vs. the control group, although 4/18 patients with RBD and 2/10 controls had verbal encoding scores lower than 14/16, which is a level that is considered abnormal. In addition, 6/18 patients with RBD (and no controls) had a score lower than the expected limit for age for at least one of the three immediate free recall tests. The delayed (20 min) free and cued recall scores were the same between the groups. Furthermore, there was no difference in the emotional perception of the 4 texts between groups, except that patients with RBD found the positive text (“reunion”) less positive than controls (data not shown). However, the negative to-be-learned texts were perceived as equally negative by both groups.

**Table 3:** Cognitive tests in patients with REM sleep behavior disorder and control subjects

Subjects	**REM sleep behavior disorder**	**Controls**	**p-value**
Number of subjects	18	10	
MoCA Total, 0-30	26.1±2	29.2±1.1	**0.0006**
Verbal encoding, 0-16	14.8±1.8	14.6±1.3	0.44
RL1, 0-16	7.1±1.9	8.2±1.8	0.24
RL2, 0-16	8.8±2.6	10.2±1.9	0.20
RL3, 0-16	9.5±2.6	10.7±2.2	0.36
Verbal learning free recall, 0-48	25.4±6.6	29.1±5.2	0.17
Verbal learning total recall, 0-48	43.7±5.3	46.6±2	0.10
Delayed verbal free recall, 0-16	11.3±2.7	12.2±1.5	0.50
Delayed verbal total recall, 0-16	14.8±2.2	15.7±0.7	0.36

RL1: recall phase 1; RL2: recall phase 2; RL3: recall phase 3; MoCA: Montreal Cognitive Assessment. Data were expressed ad the mean±SD.

### 4: Sleep-related memory consolidation in patients with RBD compared to the control subjects

**Figure 2:**
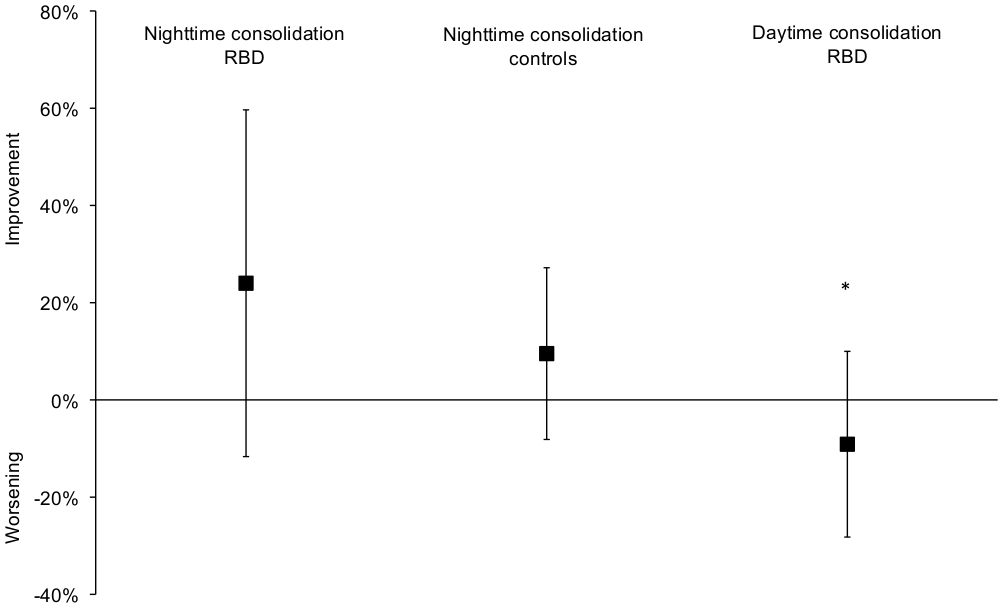
Night and day-time consolidation of patients with REM sleep behavior disorder and the controls. * p<0.005 for differences within ten nine patients who had night and day-time consolidation.

 Both groups improved their scores on the verbal tests on the morning after one night of sleep, compared to the previous evening (Figure 2, Table S1), and there was no difference between the groups. With regards to the memory consolidation in the 9 patients with RBD who were tested after daytime and night-time consolidation, the performance worsened from the morning to the next evening with a mean of -9%±19 (daytime consolidation) but it improved from the evening to the morning with a mean of +29%± 45 (*p*=0.03, Wilcoxon rank test). 

Sleep measures (including sleep onset latency, REM sleep onset latency, total sleep time, sleep efficiency, duration of wakefulness after sleep onset and of each sleep stage, arousal index, apnea-hypopnea index, periodic leg movement index) were not associated with the level of sleep-related memory changes in the 28 participants. Importantly, there was strong sleep-related verbal consolidation in the two patients with RBD and dementia criteria who were excluded from the analysis 1 patient showed an increase of 210% in sleep-related verbal consolidation (from 20 recalled words during the previous evening to 62 recalled words in the morning) and the other patient demonstrated an increase of 324 % (from 17 words to 72 words). However, all other measures (demography, sleep and cognition) did not change the significance of these results (data not shown). 

### 5: Sleep talking material and verbal replay

Eleven patients with RBD spoke during REM sleep, with verbal utterance including 1 to 101 words (median 20 words), and 0 to 21 sentences (median 3 sentences) per patient. The utterances are fully detailed in Annex 2 (French language and English translation by an accredited translator). The total time with spoken language during sleep in the whole sample was 3 min, hence 0.003% (3 chances per 10,000) of sleep with utterance. The two independent judges discarded the verbatim obtained in REM sleep in 10 of the 11 patients, with no evidence of literal or semantic replay of the learned material. They both considered that the series of sentences uttered by Patient 2, including precisely this sentence: “You’re a little slut because you go hanging about in the streets” was semantically related to fragments of the first text (“A love to death”) learned during the previous evening (Video 1), as the text related to the story of a poor mother wandering the Chicago streets looking for a job, who eventually killed her baby. This patient had a 21% rate of sleep-related consolidation with this story (from 161 to 195 words) and uttered this sentence the first night after learning the story.

## Discussion

### 1: General findings

The sleep related consolidation for the two verbal tests was maintained in patients with RBD and in the controls. The post-sleep performance was unrelated to the sleep measures (including continuity, stages, fragmentation and apnea-hypopnea index). The verbal memory worsened from the morning to the evening but improved during the night in a subgroup of 9 patients with RBD, who were tested during night-time and daytime conditions. Eleven subjects with RBD spoke during REM sleep. Among these patients, one patient pronounced a sentence that was judged semantically, but not literally, related to the text learned prior to sleeping. 

### 2: What is known about sleep-related consolidation in neurological diseases?

This is the first study that investigated sleep related verbal consolidation in parasomnia, and more specifically in RBD. Previous groups have studied sleep-dependent memory consolidation in other sleep disorders including primary insomnia, narcolepsy-cataplexy and obstructive sleep apnea [13,34–37]. Patients with primary insomnia have impaired sleep-dependent consolidation of verbal and visual declarative information, while procedural memory (mirror tracing) was equivocally affected. Efficient declarative memory correlated with higher N3 sleep time in patients with insomnia [34], while here patients with RBD had normal sleep-dependant memory consolidation despite lower N3 sleep time than controls, and no correlation between performance and N3 sleep time. Patients with moderate obstructive sleep apnea syndrome demonstrated impaired verbal declarative memory consolidation. Patients with narcolepsy displayed impaired consolidation of visual procedural skills. We previously found that a procedural task (a variant of the serial reaction time) was consolidated after night-time sleep in 20 patients with RBD [19]. Taken together, these results show that patients with RBD maintain the ability to consolidate verbal and procedural skills during sleep. This finding was even more pronounced in some patients with RBD with scores below the norm from several cognitive tests for age, sex and education level. In addition, patients with RBD usually have a lower cognitive performance compared controls, independent of the groups being compared (i.e., idiopathic RBD vs. control group or Parkinson’s disease with vs. without RBD groups) [25]. Eventually, we serendipitously tested two patients with Lewy body dementia and RBD after sleep. They demonstrated major (+210% and +324%, respectively) sleep-related verbal consolidation, despite poorly remembering the text in the evening and having no visible spindle activity during sleep. Thus, three amnesic patients learned a procedural game and produced hypnagogic images relative to the game at sleep onset of a nap after learning, despite none of the patients remembering the game or the experimenter from one session to the next [38]. The ability to enact dreaming in RBD is related to impaired a blockade of muscle tone during REM sleep, most likely due to a brainstem lesion in the REM atonia system, which does not appear to affect the upper memory consolidation systems. 

### 3: Verbal replay

The theory of sleep-related consolidation assumes that new information is somehow reactivated during sleep, with off-line reprocessing of traces at distinct levels of memory functioning [3,39]. In this study, we investigated whether the learned material could be orally reprocessed during sleep talking. We found that only one subject with RBD uttered a sentence during night-time REM sleep that was semantically related to the text he had learned the previous evening. This verbal “replay” was not literally but semantically related to the learned material. The representation of the story was not restricted to the isolated, veridical incorporation of the words, but had consisted of meaningful fragments of the experience (a “bad girl” wandering in the street) “interleaved” with other material. Although isolated, this result suggested that the sleeping brain incorporates some elements of a recently learned story within its mental content, suggesting that not an exact replay but rather some creative activity based on recent acquisitions. Thus, subjects that were trained on the motor task of a ski simulation incorporated some elements of this task within their mental content during the next sleep [40,41]. However, during sleep, the nature of this cognitive “replay” effect became more abstract from the original experience over time. Whether the verbal incorporation into an enacted dream content observed here reinforces the sleep-related memory consolidation is still unknown; however, this patient demonstrated a good percentage (+21%) of consolidation. It has been previously shown that pre-sleep verbal stimuli retained for further recall are often incorporated into REM sleep-associated dream contents [42]; however, these previous studies were based on dream reports, which were differentially biased (recall bias, will to please the investigator, censure). This is the first time that the learned material is directly online and visible to the investigators during sleep, unbeknownst to the sleeper himself. 

### 4: Limitations of the study

This study has some limitations. First, the size of the sample (and particularly the control group) was limited, and the RBD group had a mix of both idiopathic and Parkinson’s disease-associated RBD. However, we aimed to test consolidation in the RBD model to observe verbal replay. Patients with RBD may have impaired daytime cognition. We circumscribed this bias by restricting the analysis to subjects with a MoCA score greater than or equal to 23. Moreover, only one patient uttered a sentence that was semantically related to the learned material. Importantly, sleep talking is rare and short, even in patients with RBD. The sleep talking material was obtained only in 11 out of 20 patients, with a median of 3 sentences per sleeper. This material lasted from a few seconds to a maximum of two minutes of sleep talking, i.e., less than 0.003% of sleep with overt speech (3 chances over 10,000 in a sample of 20 patients), which limits the accessibility of the brain’s verbal processing during sleep. Despite this extremely low probability of observing any replay, we obtained evidence of partial replay in one patient among 18, which makes this single observation valuable. Interestingly, this patient was the greatest sleep talker of the group, which may have facilitated this observation. 

## Conclusion

In conclusion, we showed for the first time that verbal declarative memory normally consolidates during sleep in patients with RBD, including patients with dementia. In addition, one patient demonstrated that the learned material was incorporated, at least at the semantic level, during sleep talking, unbeknownst to the sleeper himself. This overt evidence provides some new insight into the creative activity of the sleeping brain. 

## Supporting Information

Annex S1 **Texts for verbal learning.**
(DOC)

Annex S2 **Sleep talking of patients with RBD in French and English.**
(DOC)

Table S1 **Night-time, day-time and FCSRT consolidation of patients with REM sleep behavior disorder and controls.** ^b^p<0.05 for a difference with night-time consolidation. NA: not applicable, not performed.
(DOC)

Video S1 **Video of the patient with RBD who semantically rehearse part of the learned text.**
(MP4)
