# Supplementary material for: Sleep-Related Declarative Memory Consolidation and Verbal Replay during Sleep Talking in Patients with REM Sleep Behavior Disorder
Source: PLoS One. 2013 Dec 13;8(12):e83352. doi: 10.1371/journal.pone.0083352 (PMC3862769; doi:10.1371/journal.pone.0083352)
Supplement: Annex S1 — Texts for verbal learning. (DOC) [file pone.0083352.s001.doc]

These texts have been translated from French into English by an accredited translator.

**A LOVE TO DEATH**

The day Mary Stastch killed her baby, Chicago’s nineteenth ward reeked of overripe fruit and kerosene. According to the *Chicago Tribune* of July 29, 1911, the unemployed single mother and recent immigrant from Austria left Cook County Hospital two days earlier and “wandered about Chicago with the baby in her arms, looking for work”. However, with the growing labor crisis, which left nearly 250,000 people jobless, her search would have been difficult even without a newborn in toe.

As if it wasn’t enough, the day after, 350 policemen raided the largely immigrant neighborhood of Maxwell Street, causing what newspapers described as “a day of rioting and wild disorder that had not been seen in Chicago since before”. Wagons were overturned, grocery store windows smashed and fruit carts were doused with fuel in a desperate struggle between peddlers, police and strikebreakers.

In the unreal silence that followed, Mary Stastch quietly strangled her infant. Cradling the dead child in her arms, she then carried its body several miles away to where it was later discovered, hidden behind garbage in Chicago’s outskirts.

**CANNIBALISM**

The meeting turned into a horror movie. A 30 year-old Russian girl gets acquainted with her victim, age 28, via an Internet meeting site. On August 19^th^, she invites him home, stabs him to death, and cuts his body into pieces, lingering over the cutting of his penis, in order to eat him. For a week, she cooks his remains; making steaks, meatballs and sausages. The woman was arrested by the Murmansk police, northwest of Russia.

In France, the last resounding affair of cannibalism goes back to June, 2012. A prisoner was condemned to a 30-year prison sentence, with a minimum recommendation of 20 years, for the murder of a cellmate on January, 2007 and for eating a piece of his lungs. During the night between January 2 and January 3, 2007, Nicolas Cocaign, a man with short hair and tattoo markings on his face, shared a cell at the Bonne-Nouvelle prison of Rouen with two other prisoners. He struck one of them, Thierry Baudry, over a hygiene quarrel, before eventually choking him to death. He then opened the thorax of his victim using a razor blade and carved out an organ, mistaking it for the heart. In fact, he had dissected out a piece of lung. He ate part of the lung raw and then cooked the rest. “What I did, I liked doing », has declared Nicolas Cocaign to experts.
