# Supplementary material for: Sleep-Related Declarative Memory Consolidation and Verbal Replay during Sleep Talking in Patients with REM Sleep Behavior Disorder
Source: PLoS One. 2013 Dec 13;8(12):e83352. doi: 10.1371/journal.pone.0083352 (PMC3862769; doi:10.1371/journal.pone.0083352)
Supplement: Annex S2 — Sleep talking of patients with RBD in French and English. (DOC) [file pone.0083352.s002.doc]

These texts have been translated from French into English by an accredited translator. The total time with spoken language during sleep over the whole group is 178 s (<3 min).

Patient 1: French : "C'est pas grave" ; "On entend pas". (7 words, 2 sentences, 4 seconds of spoken language)

English translation: "It doesn’t matter" ; "I Can’t hear well".

Patient 2: French: "Tu te fous pas de ma gueule comme ça … Tu es restée m'attendre où ? Faut m'expliquer ça hein ? Je veux maintenant des explications t'es une petite salope parce que tu vas trainer dans les rues ... et tu viens ... tu sais je te connais hein ?... je te connais" ; "A ce niveau-là … je vous emmerde … avec tes problèmes c'est mon problème, c'est ton problème, tu te démerdes … si tu n'es pas content tu t'en vas … tu m'as presque menacé ... faut pas refaire ça parce que je suis le maitre chez moi" ; "Oui?" ; "Hein" ; "Restez-là". (101 words, 21 sentences, 82 s of spoken language)

English translation: "Don’t put me on like this…where did you wait for me? You must explain this, eh? I want an explanation now, you’re a little slut because you go hanging about in the streets…and you come…I know you very well, you know?...I know you” ; " at this level…to hell with you…with your problems, it’s my problem, it’s your problem, you get out of your own bloody mess…if you’re not happy, go away…you nearly threatened me...don’t do this again because I’m in charge round here" ; "Yes?" ; "What" ; "Stay here".

Patient 3: French : "Attention" ; "il a pas tort, moi je veux chacun en aura" ; "cinq ans ... et sans ce putain de général ils sont tous des esclaves puisque vous continuez à faire il adore ça toute seule dans le pays merde voilà l'avantage … alors ne vous pressez pas les gamins vous avez tort". (51 words, 8 sentences, 21 s of spoken language)

English translation: "Careful" ; "He’s not wrong, I want and everyone will have some" ; "Five years...and without this goddamn general they all are slaves and because you go on doing what he loves it alone in the country shit here’s the advantage…so kids do not hurry up you’re wrong".

Patient 4: French: "Vous m'avez". (3 words, 1 sentence, 1.4 s of spoken language)

English translation: "You have me".

Patient 5: French: "Hein?" ; "Eh toi tu t'en vas pas hein? Tu rigoles hein? T'as bien compris là hein?" ; "Anna? Anna". (20 words, 3 sentences, 10 s of spoken language)

English translation: "What?" ; "And you’re not going, eh? You’re laughing, eh? You’ve understood it, eh?" ; "Anna? Anna".

Patient 6: French: "Tomber … poisson" ; "Psst, psst" ; "J'aime pas". (7 words, 2 sentences, 7 s of spoken language)

English translation: "Falling … fish"; "Psst, psst" ; "I don’t like it".

Patient 7: French : "C'est … ou … sont toutes les et nous les avons présentées … nous les avons présentées les opérations maintenant euh je je le raisonnement le long de mon expérience parce que il faut crédibiliser ce que je vous dis … et qu'est-ce qui arrive la lumière". (44 words, 7 sentences, 30 s of spoken language)

English translation: "It’s…or…there all the and we have introduced them…we have introduced the operations now or I … the reasoning along my experience because we have to give credibility to what I’m saying and what is happening the light".

Patient 8: French: "Coco". (1 word, no sentences, 1 s of spoken language)

English translation: "Darling".

Patient 9: French : "Aucune des deux" ; "Je ne vois pas rien n'empêche … je ne vois pas où est le problème le jour où j'ai à faire le matériel" ; "T'as fini oui ou non?". (33 words, 5 sentences, 13.4 s of spoken language)

English translation: "None of both" ; "I can’t see nothing stopping it … I don’t see where the problem is the day I must make the equipment" ; "Have you finished or not ?".

Patient 10: French : "Ah, s'il vous plait ah non on peut rien quand on est en vacances c'est pas pour ça que c'est un drame, poulette … ah". (27 words, 4 sentences, 7 s of spoke language)

English translation: "Ah, please oh no we can’t do anything when we’re on holiday it’s not the end of the world, my chick…ah".

Patient 11: French: "Minable". (1 word, no sentences, 1 s of spoken language)

English translation: "Pathetic".
