# Supplementary material for: Sleep-Related Declarative Memory Consolidation and Verbal Replay during Sleep Talking in Patients with REM Sleep Behavior Disorder
Source: PLoS One. 2013 Dec 13;8(12):e83352. doi: 10.1371/journal.pone.0083352 (PMC3862769; doi:10.1371/journal.pone.0083352)
Supplement: Table S1 — Night-time, day-time and FCSRT consolidation of patients with REM sleep behavior disorder and controls. bp<0.05 for a difference with night-time consolidation. NA: not applicable, not performed. (DOC) [file pone.0083352.s003.doc]

| Patients | **REM sleep behavior disorder** | **Controls** | **p-value**  **(RBD vs. controls)** |
| --- | --- | --- | --- |
| Night-time consolidation,% | 24.1±35.6  n=18 | 9.4±17.7  n=10 | 0.30 |
| Night-time consolidation,%  Subgroup tested during night-time and daytime | 28.9±44.6  n=9 | NA | NA |
| Day-time consolidation,% | -9.1±19.1^b^ | NA | NA |
| Night-time consolidation of the FCSRT,% | 22.6±21.9 | 23.8±12.3 | 0.86 |
